# The Synthesis of Polycarboxylate Dispersants Containing Benzenesulfonic Acid Groups and Their Performance in Promoting Coal Particle Dispersion

**DOI:** 10.3390/molecules30122493

**Published:** 2025-06-06

**Authors:** Lin Li, Zhisen Li, Shuo Yang, Chuandong Ma, Wenqi Zhang, Meng He, Xiaofang You

**Affiliations:** 1 College of Energy and Mining Engineering, Shandong University of Science and Technology, Qingdao 266590, China; 2 Coal Product Research and Development Management Center, Xinwen Mining Group Co., Ltd., Tai’an 271200, China; 202181090001@sdust.edu.cn

**Keywords:** polycarboxylate, coal water slurry, dispersant, adsorption

## Abstract

In this study, a polycarboxylate coal–water slurry dispersant (SSPA) containing benzenesulfonic acid groups was synthesized using allyl alcohol polyoxyethylene ether 500, sodium styrenesulfonate, and acrylic acid as raw materials. The effects of SSPA and a commercially available naphthalene-based dispersant (MF) on the slurry characteristics of low-rank coal were compared, and the maximum solid content of CWS prepared with SSPA reached 65.2%, which was 4% higher than that achieved with MF (61.2%). Unlike the more electronegative MF dispersant, SSPA features long polyether side chains that exert a robust steric hindrance effect, significantly enhancing coal particle dispersion. This results in a decrease in apparent viscosity and an increase in the stability of the CWS formulated with SSPA. Furthermore, adsorption experiments revealed that the adsorption kinetics of both SSPA and MF on coal conformed to the pseudo-second-order kinetic model. SSPA’s adsorption on coal particles followed the Langmuir isothermal adsorption model, and the *K*_L_ value of 0.0094 for SSPA was greater than that of MF (0.0086). This indicates that SSPA has a stronger affinity for the coal surface. Overall, the superior adsorption efficacy of SSPA is attributed to the benzene ring in its nonpolar group, which facilitates steric hindrance with aromatic structures in coal. Additionally, SSPA improves slurry stability, achieving a penetration rate of 96.7%. Finally, the carboxylic acid groups in SSPA likely engage in electrostatic attraction with cations on the coal surface, enhancing adsorption.

## 1. Introduction

Coal remains a critical energy resource in China [1], and its efficient and clean utilization is essential for environmental protection [2]. Coal water slurry (CWS), a coal-based liquid fuel, emerged in the 1970s [3]. Due to its low combustion temperature, CWS can reduce pollutant emissions during combustion. Additionally, owing to its excellent fluidity and stability, CWS has gained significant popularity within the coal chemical industry. China is abundant in low-rank coal (LRC) reserves, and because of its low price, high volatile content, and other characteristics, China mainly uses LRC to prepare CWS. However, the performance of LRC slurries is limited due to their low degree of metamorphism, high oxygen-containing group content, and numerous pores [4,5]. Given this, improving the slurry concentration of LRC has become a popular research focus for scholars [6,7].

High-performance dispersants can significantly improve the slurry characteristics of LRC. The primary dispersants employed in CWS include cationic, anionic, and non-ionic [8]. Due to their low performance and high cost, cationic dispersants are rarely used [9]. Nonionic dispersants do not dissociate in an aqueous solution; instead, they mainly rely on the steric hindrance effect to enhance the dispersion effect of particles. However, the dispersion performance is poor, the cost is high, and they have not been widely used. In contrast, the dispersion ability of anionic dispersants is outstanding. After adsorption, they can increase the negative charge of coal particles and have a strong steric hindrance effect, which can effectively improve the suspension of coal particles. Therefore, they are widely used in the production of CWS. The types of anionic dispersants include humic acid, lignin, naphthalene, and polycarboxylic acid. Humic acid is rich in nature, and its basic structure is composed of heterocycles, a small number of aliphatic rings, and many aromatic rings [10]. Because of its similar molecular structure to coal, there is an excellent affinity between them. However, the compound’s solubility in water is low, particularly in neutral and acidic conditions and other solvents. Lignin is a natural renewable resource, and the lignosulfonate prepared via sulfonation is more widely used in preparing CWS, but it has a defect of a low slurry concentration [11,12]. Naphthalenesulfonate dispersants are mainly prepared via the polycondensation of naphthalene sulfonate and formaldehyde [13]. The advantages of the naphthalene system are low viscosity and excellent rheological properties, but the slurry prepared using this kind of dispersant is prone to precipitating. Carboxylate dispersant [14] is a promising type of CWS dispersant with an excellent slurrying effect. It is environmentally friendly, has a flexible structure, and has many applications. Common polycarboxylic acid dispersants consist of a non-polar main chain, a polyether side chain, and a carboxyl group [15]. The performance can be further enhanced by introducing different characteristic groups, and a performance dispersant improves the slurry performance of the LRC.

In this study, allyl alcohol polyoxyethylene ether 500 (APEG-500), sodium styrenesulfonate (SSS), and acrylic acid (AA) were selected as functional monomers to design and synthesize polycarboxylate dispersants with styrenesulfonate groups (SSPA). The synthesized polycarboxylic acid dispersant was utilized to prepare a CWS to investigate the dispersant’s effect on the slurry formation performance. Compared with conventional dispersants such as MF, the synthesized SSPA incorporates benzenesulfonic acid groups and polyether side chains, which enhance adsorption ability, electrostatic repulsion, and steric hindrance, thereby improving the dispersion efficiency of coal particles in slurry systems. Furthermore, the mechanism of the dispersant’s action to promote the dispersion of coal particles was subjected to further investigation.

## 2. Results and Discussion

### 2.1. Characterization of Dispersant Structures

#### 2.1.1. FT-IR Spectral Analysis

**Figure 1:**
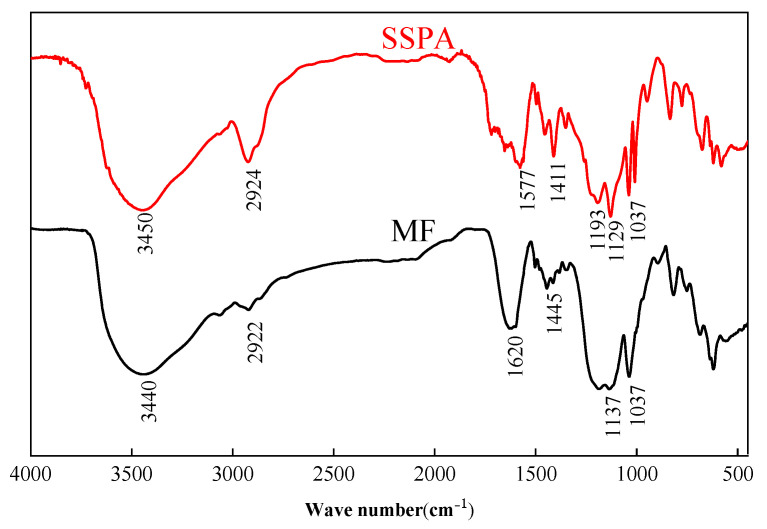
The FTIR spectrum of SSPA. Peaks at ~1577 cm⁻^1^ indicate benzene ring vibrations, those at ~1193 cm⁻^1^ correspond to ether bonds, and those at ~1700 cm⁻^1^ represent carboxyl group stretching.

Figure 1 shows the FTIR scanning spectra of SSPA and MF.

In Figure 1, 1441~1577 cm^−1^ indicates the skeleton stretching vibration absorption peaks of the benzene ring, 1411 and 1129 cm^−1^ are the characteristic absorption peaks of -${SO}_{3}^{-}$, 1193 cm^−1^ are the absorption peaks of poly (oxyethylene ether), and the C=C bond from 1625 to 1680 cm^−1^ basically disappears, which indicates that the acrylic acid and other monomers were fully incorporated during the polymerization reaction. In MF, the characteristic absorption peaks at 1448 and 1037 cm^−1^ indicate the -${SO}_{3}^{-}$ group, and the absorption peaks for the naphthalene ring skeleton stretching vibrations are between 1444 and 1620 cm^−1^. This indicates that MF primarily contains sulfonic groups and naphthalene ring structures.

#### 2.1.2. Nuclear Magnetic Hydrogen Spectroscopy Analysis

**Figure 2:**
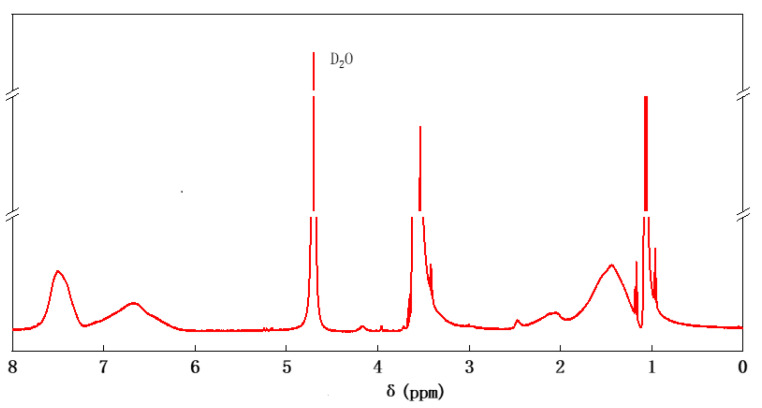
The ^1^H NMR spectrum of SSPA. Chemical shifts at δ = 3.35–3.73 correspond to polyether side chains, those at δ = 6.45–7.75 correspond to aromatic protons, and the disappearance of vinyl protons (δ = 5.0–6.0) confirms successful polymerization.

Figure 2 shows the ^1^HNMR spectrum of SSPA with D_2_O as a solvent.

As shown in Figure 2, δ = 4.73 is the solvent peak, and δ = 1.14 to 1.86 are the chemical shifts in the hydrogen in the molecule’s sub-methyl structure. The disappearance of vinyl proton signals (δ = 5.0–6.0 ppm) confirms successful acrylic acid polymerization. δ = 3.35 to 3.73 indicates the chemical shift in the hydrogen in the -CH_2_CH_2_O- structure of the side group polyether chain. δ = 6.45 to 7.20 and 7.32 to 7.75 are the hydrogen chemical shifts on the benzene ring. Meanwhile, the double-bond characteristic proton peak (δ = 5.0 to 6.0) largely disappears. The above results indicate that the synthesized polymers had structures such as benzene rings and polyether chains and that the carbon–carbon double bonds in the monomers used for the experiments were involved in the reaction and were more fully reacted.

#### 2.1.3. Molecular Weight Distribution Analysis

GPC can analyze the molecular weight distribution of an agent and is used to determine the molecular weight of a synthesized agent. The results of the molecular weight determination of SSPA are shown in Table 1.

In Table 1, the heavy average molecular weight of SSPA is 13,504 g/mol. The distribution coefficient of 1.43 indicates that the synthesized dispersant was more concentrated. SSPA has a large molecular structure and contains anionic functional groups, which can effectively form steric hindrance and electrostatic effects. A lower Mw/Mn (1.43) indicates a relatively narrow molecular weight distribution. This contributes to uniform performance and predictable behavior in slurry systems, which is more conducive to the suspension and dispersion of coal particles.

### 2.2. Analysis of Dispersion Performance

#### 2.2.1. Dosage of Dispersant

**Figure 3:**
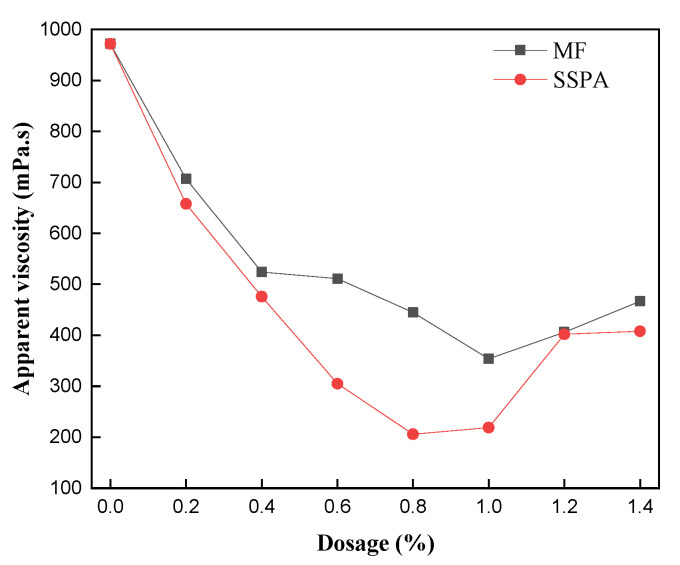
The apparent viscosity of CWS as a function of the dispersant dosage.

The solid mass fraction of the slurry was set to 60%. The variation in the slurry’s apparent viscosity (Va) with an increasing amount of dispersant was measured and is shown in Figure 3.

In Figure 3, it is shown that the viscosity of the slurry formed by the two dispersants showed a tendency to decrease and then to increase, with the lowest viscosity of 354 mPa·s when the addition of MF was 1.0 wt% and the lowest viscosity of 206 mPa·s when the addition of SSPA was 0.8 wt%. SSPA exhibited a significant viscosity-reduction effect. For the same amount of dispersant, the viscosities of CWS prepared with the SSPA dispersant were lower than those of CWS prepared with MF. The dispersant enhances the hydrophilicity of the coal surface and simultaneously reduces the agglomeration effect of coal particles due to electrostatic force, which increases the fluidity and reduces the Va. However, excess dispersant causes the coal surface to bind many water molecules and form a thicker hydrated film. There is a reduction in free water in suspension systems, so inter-particle collisions increase, increasing slurry viscosity [16]. The reason for the superior viscosity-reducing performance of the SSPA dispersant is that SSPA carries a long side-chain group that can provide spatial protection to reduce the agglomeration of coal particles. It also contains carboxyl groups that can be combined with the hydrophilic groups on coal to enhance the adsorption of SSPA. Furthermore, the benzene ring structure on SSPA is subjected to the π-π stacking effect, which further enhances its binding ability with the coal surface. Therefore, the SSPA dispersant has a better dispersing and viscosity-reducing effect.

#### 2.2.2. Maximum Solid Content

**Figure 4:**
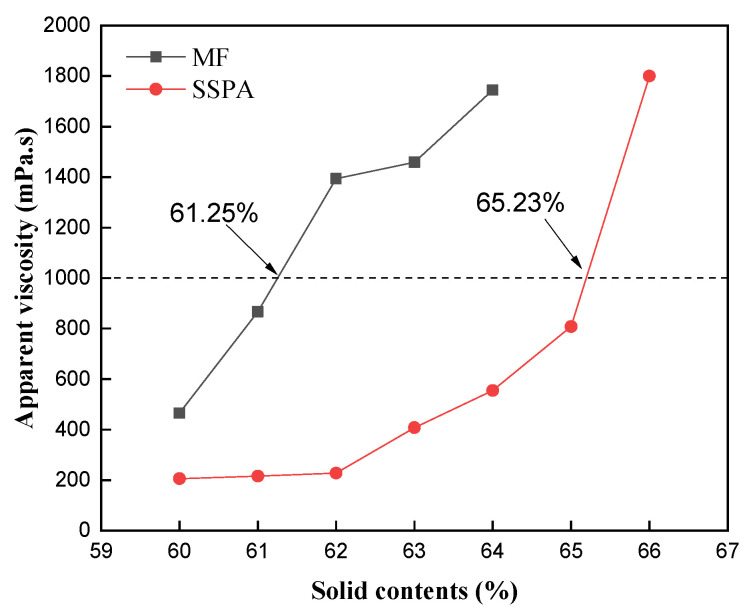
The maximum solid contents.

The optimum value of dispersant dosage was selected. The variation in Va in CWS with the increase in its solid mass fraction was determined at 25 °C and a shear rate of 100 s^−1^, and the results are shown in Figure 4. The dosages used were 0.8 wt% for SSPA and 1.0 wt% for MF, as determined previously.

As shown in Figure 4, the Va of the slurry gradually increased when increasing the solid mass fraction of the CWS, and the maximum slurry-forming concentrations of the CWS prepared using the two groups of dispersants were 65.23% (SSPA) and 61.25% (MF), respectively. These values correspond to the apparent viscosity reaching 1000 mPa·s, which is indicated by the dashed line in Figure 4. This threshold is commonly used as the criterion for determining the maximum slurry concentration, representing the critical state where the slurry maintains both high solid content and acceptable flowability. The slurry concentration prepared using the SSPA dispersant was significantly higher than that of MF. SSPA exhibits excellent pulping performance. Highly valent metal cations in coal, such as Ca^2+^, make some areas of its surface positively charged. The carboxylic acid monomers in SSPA can be chelated and adsorbed with metal cations on coal. The adsorption capacity of the dispersant is enhanced, which improves the dispersion of coal particles in the solution.

#### 2.2.3. Rheological Analysis

**Figure 5:**
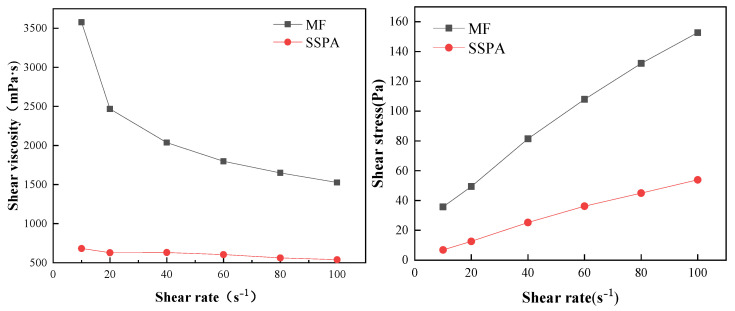
The rheological properties.

Figure 5 shows the viscosity curve as a function of the shear rate for a slurry with a concentration of 64%. The high yield stress values observed are consistent with those reported for dense coal–water slurry systems in prior studies. They reflect the substantial interparticle network and internal resistance to deformation under low shearing, which is typical for slurries with a high solid content.

As shown in Figure 5, the viscosities of both groups of CWSs gradually reduced with the increase in the shear rate, showing the characteristics of shear thinning. The slurries prepared with both dispersants meet the requirements of rheological properties for industrial applications of CWS. The rheological behavior of CWS is closely related to the dispersion state of coal particles and the steric/electrostatic interactions introduced by dispersants. A comparable influence of Si/Al ratio on rheology was also observed in alkali-activated cementitious materials, indicating the universal importance of composition–microstructure interactions in determining yield stress and viscosity [17]. The shear stress value of the slurry with SSPA was lower, indicating that the viscosity-reduction effect of SSPA is better.

The parameters obtained from the Herschel–Bulkley (H-B) model [18] fit are shown in Table 2. The fitting constants, R^2^, were above 0.999, indicating that the rheological properties of both sets of CWSs conformed to the H-B rheological model. The yield stress of CWS made using SSPA dispersant was 3614 mPa and that of CWS made using MF dispersant was 9506 mPa. The slurry produced with SSPA dispersant had a low yield stress, *τ*_0_, which made it easier to overcome the initial stress and achieve better dispersion. The flow characteristic index *n* values were all less than 1, indicating that the slurries were pseudoplastic fluids. The consistency coefficient *K* of SSPA slurry was 1513 mPa·s*^n^*, much smaller than MF slurry’s 4294 mPa·s*^n^*. This indicates that the viscosity of the slurry prepared using SSPA was lower under the same solid content condition.

#### 2.2.4. Analysis of Stability

**Figure 6:**
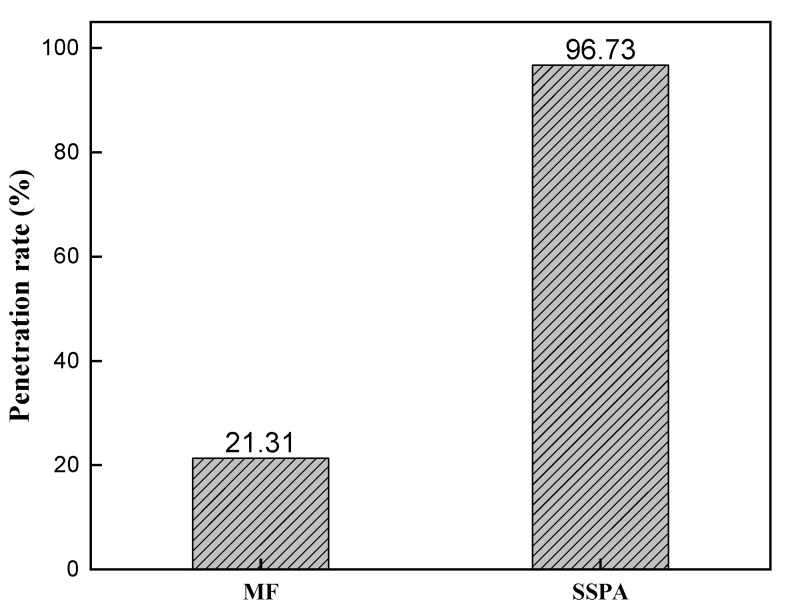
The stability of slurries.

Figure 6 shows the stability data of the slurry determined using the falling bar method. The solid content of the slurry was 64%, and the dispersant dosages were all optimal.

As shown in Figure 6, the penetration rate of MF was 21.31%, while SSPA’s was 96.73%. The stability of SSPA slurry was much greater than that of MF slurry, and the stability of the slurry was improved by using SSPA pulping. This is due to the hydrophobic main chain and benzene ring structure in the SSPA molecule that can be adsorbed with the nonpolar part of the coal particle surface, and the carboxyl group binds with the metal cation, which gives SSPA a strong ability to bind with coal. In contrast, MF interacts with the nonpolar portion of the coal mainly through the aromatic ring portion. In addition, SSPA enhances the electrostatic repulsion of coal particles. Moreover, the polyether groups of SSPA can enhance the steric hindrance effect between coal particles, further promoting particle dispersion.

It is worth noting that this study was conducted under neutral-pH conditions and at room temperature (25 °C). The effects of pH, temperature, and potential toxicity were not investigated in this work. They will be addressed in future studies to assess the environmental adaptability and safety of the dispersant.

### 2.3. Mechanism Analysis

#### 2.3.1. Wettability Analysis

**Figure 7:**
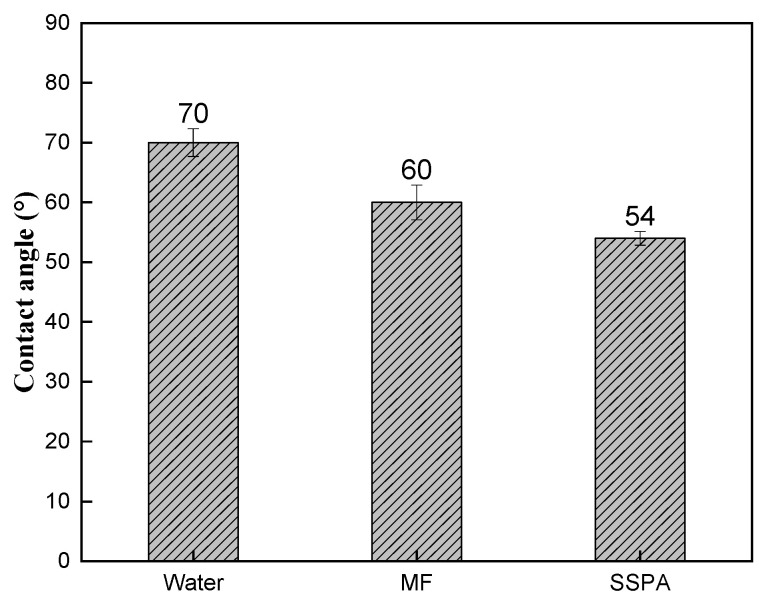
The contact angle.

Smaller contact angles indicate increased surface hydrophilicity. Figure 7 shows the contact angle data of different solutions on coal.

As shown in Figure 7, the contact angles of water, MF, and SSPA dispersant solutions on the coal surface were 70°, 60°, and 54°, respectively. The contact angle became smaller after the addition of dispersant, indicating that the addition of dispersant increased the hydrophilicity of the coal surface. This is because the dispersant adsorbs on the coal and the polar groups point outward, enhancing the hydrophilicity of the coal. The SSPA solution had the lowest contact angle, which is attributed to the presence of polar polyether side chains and carboxylic acid groups that enhance the hydrophilicity of the coal surface.

#### 2.3.2. Zeta Potential Analysis

**Figure 8:**
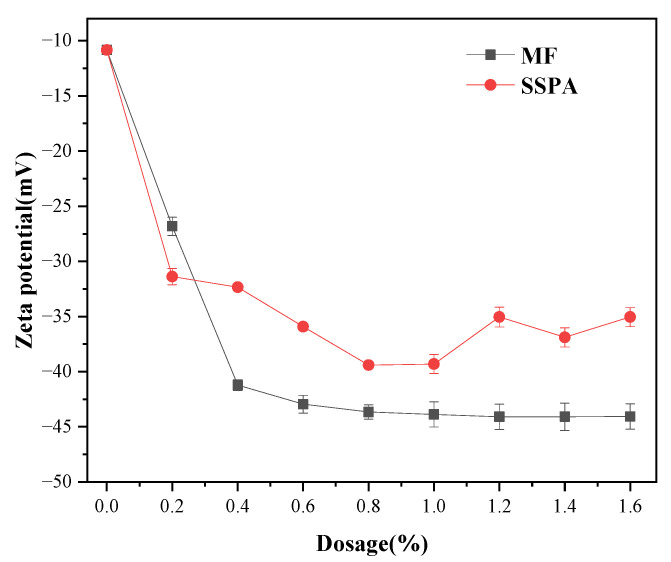
The zeta potential.

The electrostatic interactions of suspended coal particles were evaluated using zeta potential values. The higher the absolute value is, the higher the electrostatic repulsion between particles is and the more favorable the particle dispersion is [19]. Suspensions were prepared using MF and SSPA as dispersants, respectively, and the potentials on the coal in the dispersed system were analyzed. The results are shown in Figure 8.

As shown in Figure 8, the zeta potential of the original coal was −10.8 mV. The amount of negative charge on the coal surface increased rapidly with the addition of both dispersants. The lowest zeta potential was −38.0 mV in the presence of the SSPA dispersant and −44.8 mV when the MF dispersant was added to the suspension. The MF dispersant’s system is more electronegative because carboxyl groups and polyether long chains dominate the molecular structure of PA. At the same time, MF contains more sulfonic acid groups. Despite sulfonic groups typically being less electronegative than carboxyl groups, their higher density in MF contributes to a more electronegative slurry system. The results show that the viscosity of the slurry prepared using SSPA dispersant decreased significantly, mainly due to the improvement in coal surface wettability and steric hindrance, and the negative repulsion had a partial effect.

#### 2.3.3. Adsorption Performance Study

The adsorption of dispersant has an important effect on coal particles’ wettability and stacking effect [7]. Exploring the adsorption properties of dispersants on coal helps reveal the slurry formation mechanism of CWS.

(1) Standard curve.

**Figure 9:**
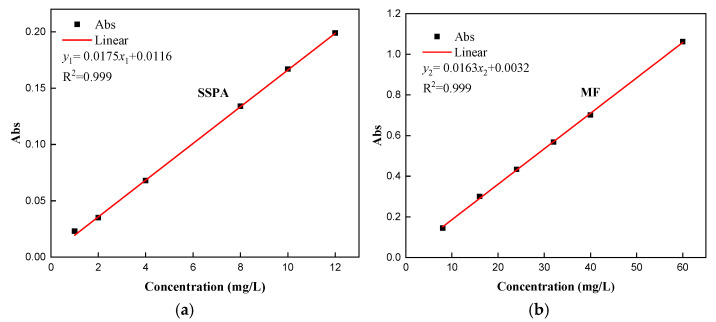
The standard curve of dispersants: (**a**) SSPA; (**b**) MF.

A series of dispersant solutions of different concentrations were configured, and the absorbance of different concentrations of dispersants was recorded to determine the relationship between absorbance and the mass concentration. The standard curves of SSPA and MF solutions plotted with the concentration as the horizontal coordinate and absorbance as the vertical coordinate are shown in Figure 9.

(2) Adsorption equilibrium and kinetic analysis.

**Figure 10:**
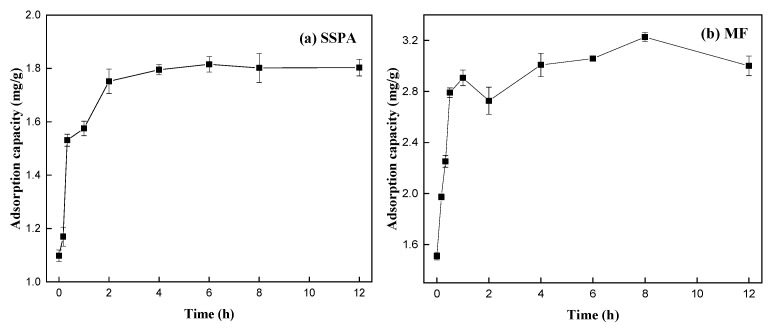
The adsorption of dispersants with different adsorption durations: (**a**) SSPA; (**b**) MF.

Figure 10 shows the variation in dispersant adsorption on coal for different adsorption durations.

As shown in Figure 10, in the early stages of adsorption, the slope of the adsorption curve is quite steep, indicating a fast adsorption rate. As the adsorption time increases, the slope of the adsorption curve decreases, with the adsorption rate slowing down. The amount of adsorption tends to stabilize after around 4 h. This is primarily because, in the initial stages of adsorption, the adsorption resistance is low and many adsorption sites are available on the particle surface. As the adsorption time increases, the number of unoccupied adsorption sites decreases, and the resistance to the dispersant entering the particle pores increases, slowing down the adsorption rate until a dynamic equilibrium is reached. The adsorption curve of the SSPA dispersant is steeper than that of MF in the initial phase, indicating that SSPA adsorbs more rapidly on coal at the beginning.

By fitting adsorption kinetic data using models, the adsorption behavior of dispersants on coal could be studied more thoroughly. The pseudo-first-order (PFO) and pseudo-second-order (PSO) kinetic models [20,21] used for fitting adsorption kinetic data are shown in Section S1.2 of the Supplementary Materials.

**Figure 11:**
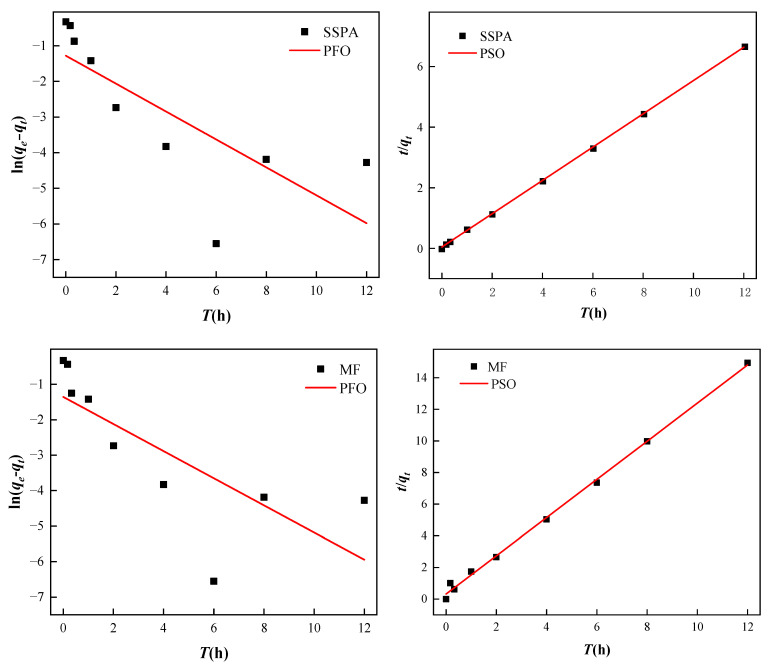
Adsorption kinetics model fitting.

Figure 11 shows a linear fit of the kinetic data for the adsorption process of SSPA/MF dispersant on the coal surface. The kinetic data for both dispersants converge to the straight line fitted by the PSO kinetic model. Table 3 shows the parameters obtained by fitting the kinetic data. The R^2^ values fitted to the PFO kinetic model were 0.5829 (SSPA) and 0.2722 (MF), and the R^2^ values fitted to the PSO kinetic model were 0.9977 (SSPA) and 0.9981 (MF), respectively. The R^2^ values of the PSO kinetic models were all higher than those of the PFO kinetic model, indicating that PSO kinetics is more suitable for describing the adsorption behavior of these two types of dispersants on coal. The adsorption process is controlled by the chemisorption mechanism. Larger values of *K*_2_ indicate a faster adsorption rate and a shorter time to reach equilibrium [22].

(3) Isothermal adsorption analysis.

**Figure 12:**
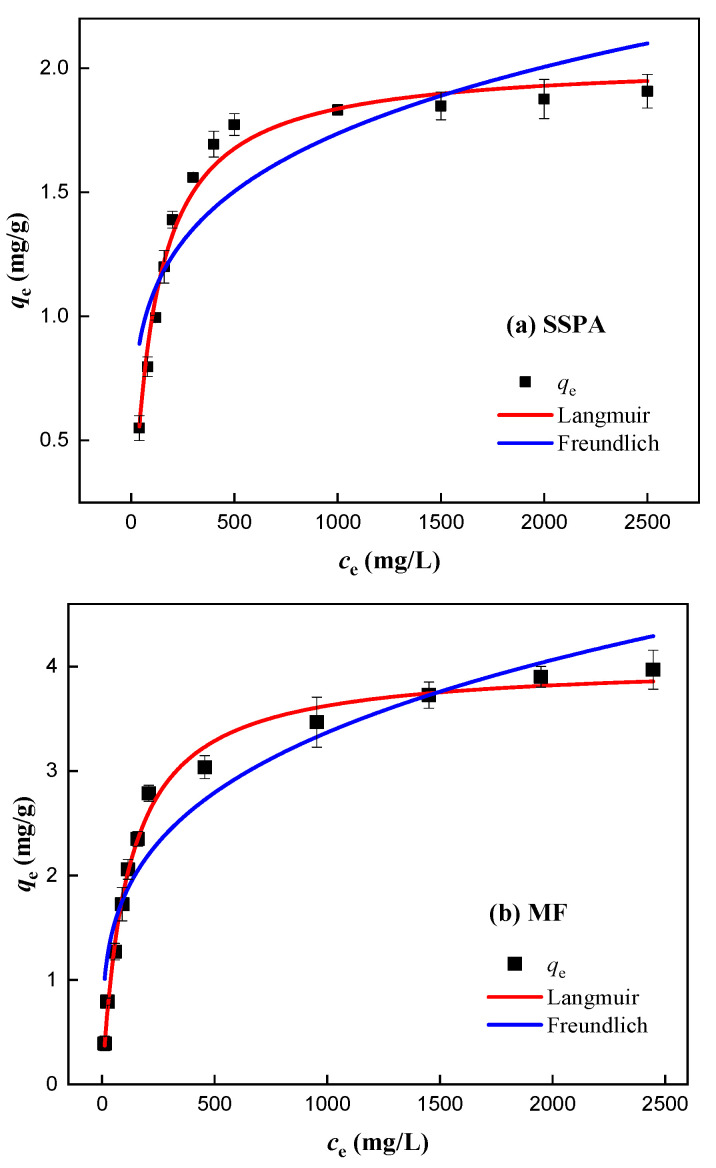
The adsorption isotherm curves: (**a**) SSPA; (**b**) MF.

The adsorption of different concentrations of dispersants on coal was determined at 25 °C. The data from the isothermal adsorption experiments and the curves fitted to the data are shown in Figure 12. In Figure 12, the equilibrium adsorption of SSPA on the particle surface increased with the increasing concentration of the SSPA solution. After the initial concentration of the solution reached 500 mg/L, the trend of adsorption increase slowed down, and the dispersant adsorption tended to be saturated.

Isothermal adsorption data are usually described using Langmuir and Freundlich models, and the fitted parameters can be used to describe the characteristics of the adsorption process and the performance of the adsorbent [23,24,25]. Section S1.3 in the Supplementary Materials details the adsorption model formula.

The parameters obtained from the model fitting are shown in Table 4.

As shown in Table 4, the isothermal adsorption data of the two dispersants were fitted using the Langmuir model, and the R^2^ was greater than 0.98, with a superior model fit. The maximum adsorption of SSPA obtained from the fitting was 2.03 mg/g, which was lower than that of MF (4.03 mg/g). The adsorption amount of SSPA was lower than that of MF because SSPA molecules have a larger molecular weight, occupying more adsorption sites during adsorption. Additionally, the carboxyl groups in SSPA can bind with metal ions on coal, occupying adsorption sites and resulting in relatively less SSPA adsorption. This also verifies that the amount of SSPA used in dispersant experiments was lower than that of MF. *K*_L_ is Langmuir’s equilibrium constant, and the magnitude of its value indicates the strength of the adsorption capacity of the dispersant [26]. The *K*_L_ value of 0.0094 for SSPA was greater than that of MF (0.0086), indicating that the adsorption capacity of SSPA is stronger. This is attributed to SSPA’s adsorption mechanism, which involves the electrostatic attraction of carboxylate groups and metal cations in addition to the nonpolar interaction, which effectively enhances the adsorption capacity of SSPA.

## 3. Materials and Methods

### 3.1. Materials

**Figure 13:**
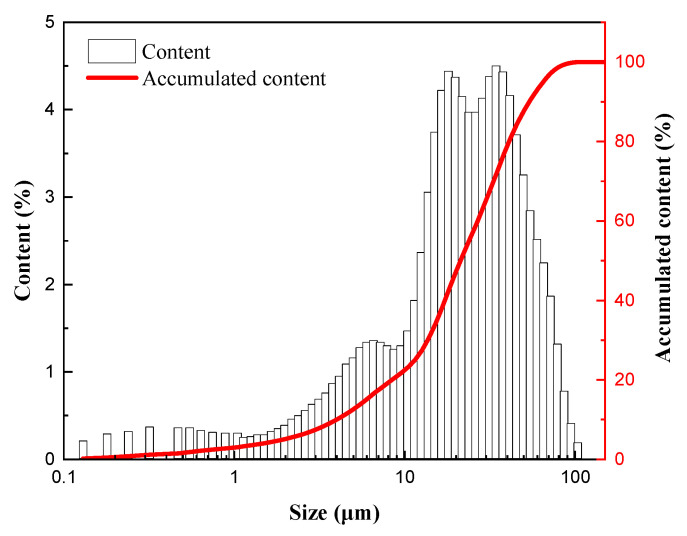
The particle size distribution.

The coal used for the experiment originated from a mine in Baotou, China. The results of the industrial and elemental analysis are presented in Table 5, and the particle size distribution is shown in Figure 13.

As shown in Table 5, the V_ad_ fraction of the coal used for the experiment is high, 29.76%, indicating that the coal exhibits characteristics of a low to medium degree of metamorphism.

Figure 13 shows that the coal used in the experiment conforms to the multi-peak particle size gradation, which has a wider particle size distribution and higher stacking efficiency than the bimodal gradation and is favorable for preparing high-concentration CWS.

### 3.2. Experimental Reagents

Sodium Salt of Polynaphthalene Formaldehyde Sulfonate (MF), analytically pure, Aladdin Reagent (Shanghai, China); Allyl Alcohol Polyethylene Glycol Ether 500 (APEG-500), analytically pure, Hai’an Petrochemical Engineering, Hai’an, China; acrylic acid (AA), analytically pure, Fuchen Chemical Reagent Co., Ltd. (Tianjin, China); sodium p-styrenesulfonate (SSS), 90%, Shaoguan Koya Fine Chemical Co., Ltd. (Shaoguan, China); sodium bisulfite (NaHSO_3_), analytically pure, Hengxing Reagent; ammonium peroxynitrite ([(NH_4_)_2_S_2_O_8_]), analytically pure, Chengdu Kelong Chemical Co., Ltd. (Chengdu, China); sodium hydroxide (NaOH), analytically pure, Tianjin Yongda Chemical Reagent Co. (Tianjin, China).

The commercial dispersant MF (analytically pure) was purchased from Aladdin Reagent. According to the supplier’s technical documentation, MF is a sodium polynaphthalene sulfonate-type dispersant with a typical average molecular weight (Mw) of approximately 8000–10,000 g/mol. Its general structure consists of naphthalene rings linked via sulfonic acid groups, forming a water-soluble polymeric surfactant.

### 3.3. Methods

#### 3.3.1. Preparation of Dispersants

**Figure 14:**
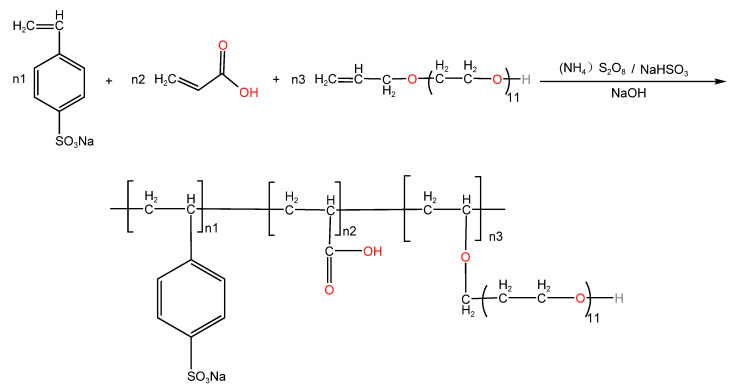
The schematic synthesis route of the polycarboxylate dispersant (SSPA). Red indicates functional groups from different monomers.

The ratio of stationary monomers was n(SSS):n(APEG-500):n(AA) = 1.0:1.0:1.5. APEG-500, SSS, sodium bisulfite, and a quantity of deionized water was added to a three-necked flask and heated to 80 °C. An aqueous solution of AA and ammonium persulfate was added dropwise via 2 dropping funnels; 1.5 h of dropping was completed, and the reaction was kept warm for 2 h after the dropping was completed. The product was cooled to 30~40 °C, and the pH was adjusted to 7~8 with a mass fraction of 30% sodium hydroxide solution. The final transparent, viscous, yellow product was SSPA. The total mass of the initiator was 4% of the sum of the masses of all monomers and n(NaHSO_3_):n[(NH_4_)_2_S_2_O_8_] = 1:1. The prepared polymer was purified with anhydrous ethanol and dried to a constant weight under warm conditions. The synthesis route of the dispersant is shown in Figure 14.

#### 3.3.2. Preparation of CWS

The experiments used dry powder to prepare CWS. The coal was crushed, passed through a 150 μm sieve, and weighed to 80 g samples, and CWS was prepared with different mass fractions. The amount of dispersant was 0.2%~1.8% of the mass of the dry base, and the slurry was produced via mechanical stirring for 5 min under a rotating speed of 1200 r/min. For the evaluation method used to determine slurry performance, please refer to Section S1.1 in the Supplementary Materials.

#### 3.3.3. Contact Angle Test

A total of 0.3 g of coal powder was taken and pressed into a flaky cylinder at 10 MPa, and the instantaneous contact angle at the coal/solution interface was determined using an optical contact angle meter. Each coal pellet was pressed at 10 MPa for 2 min to ensure consistent surface properties (DSA100, KRÜSS, Hamburg, Germany).

#### 3.3.4. Zeta Potential Test

Multiple portions of 0.2 g of coal were taken in a conical flask, and dispersants of 0, 0.2%, 0.4%, 0.6%, 0.8%, 1.0%, 1.2%, 1.4%, and 1.6% of the dry coal mass and 50 mL of deionized water were added. At 25 °C, the coal was shaken in a constant-temperature shaker for 5 h. The zeta potential of the sample was measured using a microelectrophoresis instrument (JS-94H, Shanghai Zhongchen Digital Equipment Co., Ltd., Shanghai, China).

#### 3.3.5. Determination of Characteristic Functional Groups

The KBr tablet method was chosen for the experiments, in which the samples to be tested were ground and mixed with KBr in an onyx mortar. The tablets were pressed using a tablet press at 15 MPa, and the tests were performed using a ThermoeNicolet380 (Bruker, Karlsruhe, Germany). The tests were conducted at 25 °C with a scanning range of 4000~400 cm^−1^.

#### 3.3.6. Gel Permeation Chromatography (GPC)

The dispersant solution with a concentration of 5 mg·L^−1^ was prepared with 0.1 mol·L^−1^ of NaOH solution. The GPC test was used for molecular weight detection [27] with a column temperature of 35 °C and a mobile phase of NaNO_3_ solution at a concentration of 0.1 mol·L^−1^, and the flow rate was set to 1 mol·min^−1^.

#### 3.3.7. Nuclear Magnetic Hydrogen Spectroscopy

The polymers were purified with anhydrous ethanol and dried at a low temperature. Then, hydrogen spectroscopic structural characterization of the dispersants was performed using deuterated water as a solvent.

#### 3.3.8. Adsorption Experiment

Dispersant adsorption on coal was determined using the residual mass concentration method. The maximum absorption wavelength of SSPA occurred at 231 nm, and that of MF occurred at 298 nm.

(1) Dsorption equilibrium and kinetics.

The initial concentration of the solution was 1 g/L, the dosage of the coal sample was 0.5 g, the volume of the solution was 50 mL, and the adsorption temperature was 25 °C. The adsorption times were 0~12 h. The adsorbed suspension was centrifuged at 10,000 rpm for 10 min, the upper clear liquid was taken, and then the residual coal particles were filtered out using a microporous filter membrane. The absorbance of the adsorbed solution was measured, and the mass concentration of the dispersant was calculated according to the standard curve equation.

(2) Isothermal adsorption experiment.

Isothermal adsorption experiments of dispersants were performed at room temperature. The initial concentrations were set to 0, 40, 80, 120, 160, 200, 240, 300, 400, 500, 1000, 1500, 2000, and 2500 mg/L, respectively. A total of 50 mL of the solution was taken in a conical flask, 0.5 g of coal sample was added, and the speed of the thermostatic water bath was 150 r/min.

#### 3.3.9. Stability Evaluation

The stability of the coal–water slurry (CWS) was evaluated using the falling-bar method after 24 h of static storage. Following preparation, the CWS was stored at room temperature in a glass cylinder (diameter: 3 cm; slurry height: 15 cm) and left undisturbed for 24 h. A glass rod (diameter: 5 mm; weight: 20 g) was allowed to fall freely from the top of the slurry. The rod was allowed to fall under gravity and stopped once its tip encountered a hardened sediment layer at the bottom. The penetration rate (*p*) was calculated according to the following equation:

p=h/L×100%

where h is the free-fall distance of the rod (cm), and L is the total height of the slurry (cm).

All experiments were conducted in triplicate, and the average values were used for analysis and data presentation.

## 4. Conclusions

In this study, polycarboxylic acid dispersants containing sodium benzenesulfonate were synthesized, and their performance was compared with the commercially available MF dispersant. The conclusions of the study are as follows:

This study on the coal–water slurry preparation demonstrated that the slurries produced by both dispersant groups exhibit characteristics of pseudoplastic fluids. The slurry prepared with the SSPA dispersant achieved a maximum solid content of 65.2%, surpassing the 61.2% achieved with MF. Furthermore, the substantial steric hindrance provided by SSPA effectively enhances the stability of the slurry. An analysis of the slurry formation mechanism revealed that the addition of MF and SSPA resulted in a decrease in the zeta potential of coal suspensions to −44.8 mV and −38.0 mV, respectively, thereby increasing the electronegativity of the slurry, which aids in particle dispersion. The greater electronegativity of the MF dispersant is attributed to its higher content of electronegative sulfonic groups. The kinetic fitting results for SSPA and MF adsorption to coal aligned with a PSO kinetic model, indicating that the adsorption process of these dispersants on coal is governed by chemisorption. The *K*_L_ value of 0.0094 for SSPA is greater than that of MF (0.0086). This indicates that SSPA has a stronger affinity for the coal surface, which is superior due to the combined effects of polar and electrostatic forces driving its adsorption, In contrast, MF’s adsorption primarily involves polar forces. Hence, SSPA’s adsorptive force is more robust than MF’s. In the SSPA suspension system, the electrostatic repulsion provided by the sulfonic acid group and the pronounced steric hindrance effect of the polyether long side chains facilitate particle dispersion. Conversely, MF dispersants primarily rely on electrostatic repulsion to promote particle dispersion.

While the SSPA dispersant exhibits excellent performance in coal–water slurry applications, its synthesis involves multiple monomers and purification steps, which may increase production costs. In addition, although the selected monomers have relatively low toxicity, a comprehensive evaluation of environmental toxicity has not been conducted. Future work will focus on improving synthesis efficiency and assessing the environmental safety of the dispersant.

## Figures and Tables

**Table 1:** The molecular weight distribution of dispersants.

Dispersant	Mw [g/mol]	Mn [g/mol]	Mw/Mn
SSPA	13,504	9443	1.43

**Table 2:** The fitting values of model parameters.

Dispersant	*τ*_0_/mPa	*K*/(mPa·s*^n^*)	*n*	R^2^
MF	9506	4294	0.7629	0.99907
SSPA	3614	1513	0.8280	0.99925

**Table 3:** The dynamics fitting parameters of the dispersant adsorption process.

Dispersant	PFO			PSO		
	*q*_e1_, mg/g	*K*_1_	R^2^	*q*_e2_, mg/g	*K*_2_	R^2^
PA	1.817	0.3915	0.5301	1.817	0.5498	0.9977
MF	3.23	0.2722	0.4035	3.23	0.3244	0.9981

**Table 4:** The fitting parameters of the adsorption isotherm equation.

Dispersant	Langmuir			Freundlich		
	*q*_m_, mg/g	*K*_L_, L/mg	R^2^	*n*	*K*_F_	R^2^
SSPA	2.03	0.0094	0.981	4.81	0.41	0.809
MF	4.03	0.0086	0.989	3.70	0.52	0.891

*K*_L_: 0 < *K*_L_ < 1 is favorable for adsorption, *K*_L_ > 1 is unfavorable for adsorption, *K*_L_ = 1 is linear adsorption, and *K*_L_ = 0 is irreversible adsorption.

**Table 5:** The industrial and elemental analysis.

Sample	Industrial Analysis, %				Elemental Analysis, %				
	M_ad_	A_ad_	V_ad_	FC_ad_	C_ad_	H_ad_	N_ad_	S_ad_	O_ad_
Coal	10.04	4.73	29.76	55.47	76.22	4.05	0.87	0.67	3.42

## Data Availability

Data are contained within the article and Supplementary Materials.

## Supplementary Materials

The following supporting information can be downloaded at https://www.mdpi.com/article/10.3390/molecules30122493/s1.
