# Supplementary material for: The Synthesis of Polycarboxylate Dispersants Containing Benzenesulfonic Acid Groups and Their Performance in Promoting Coal Particle Dispersion
Source: Molecules. 2025 Jun 6;30(12):2493. doi: 10.3390/molecules30122493 (PMC12196111; doi:10.3390/molecules30122493)
Supplement: Supplementary file 1 [file molecules-30-02493-s001.zip › molecules-3619481-supplementary.pdf]

Supplementary material

The Synthesis of Polycarboxylate Dispersants Containing Benzenesulfonic Acid Groups and Their Performance in Promoting Coal Particle Dispersion

Section S1.1 Slurry performance analysis

(1) Apparent viscosity measurement. The prepared CWS was mechanically stirred for 5 min to make it uniformly dispersed. The viscometer (NXS-4C, Chengdu Instrument Factory) was used to record the data six times at a shear rate of 100 s^{-1} under the condition of 25 °C, and the mean value was calculated as the apparent viscosity of the slurry.

(2) *Rheology.* The viscosities of the slurries at different shear rates were obtained and fitted using the Herschel-Bulkley (H-B) model formulation to analyze their rheological properties[27].

H-B model: $\tau = \tau_0 + K\gamma^n$……………………………(S1)

where τ is the shear stress, Pa; τ_0 is the yield stress, Pa; γ is the shear rate, s^{-1}; K is the consistency coefficient, $Pa{\cdot}s^n$; and n is the flow characteristic index (when $n>1$, the slurry is an expanding plastic fluid; when $n=1$, the slurry is a Newtonian fluid; when $n<1$, the slurry is a pseudoplastic fluid).

(3) *Maximum solids content*[3]. The maximum solids content is the concentration of slurry with an apparent viscosity of 1000 mPa·s and is usually obtained by interpolation.

(4) *Stability.* The stability of CWS was assessed by the falling rod method[15]. The CWS was stored in glass cylinders (3 cm diameter; CWS layer height 15 cm) at room temperature and left to stand for 24 hours. A glass rod (5 mm diameter, 20 g weight) was used to drop spontaneously from the surface of the CWS, stopping when the tip encountered a hard deposit. The penetration rate was calculated as follows:

$$Penetration\,rate = \frac{h}{L} \times 100 \tag{S2}$$

Where h is the free fall distance of the glass rod (cm) and L is the total height of the slurry (cm).

Section S1.2 Adsorption kinetic model

PFO kinetic model:

$$q_t = q_e \times \left(1 - e^{-K_1 t}\right) \text{ …….……………………..(S3)}$$

PSO kinetic model:

$$q_t = \frac{K_2 \times q_e^2 \times t}{1 + K_2 \times q_e \times t} \text{…………………………………(S4)}$$

where, K_1 is the PFO adsorption rate constant; K_2 is the PSO adsorption rate constant; t is the adsorption time in hours; q_t is the amount of adsorption at time t, mg/g; q_e is the equilibrium adsorption amount, mg/g.

Section S1.3 Isothermal adsorption model

The Langmuir model assumes that the adsorption process occurs on the homogeneous surface of the adsorbent[26], and its model expression is as follows:

$$q_e = \frac{k_L q_m c_e}{1 + k_L c_e} \text{………………………………… (S5)}$$

Where, q_e, equilibrium adsorption amount, mg/g; c_e, equilibrium concentration, g/L; q_m, saturated adsorption amount, mg/g; K_L, Langmuir's adsorption constant,

L/mg, characterizes the strength of adsorption capacity.

The Freundlich model is used to describe the adsorption behavior that occurs on non-homogeneous surfaces[25] with the model expression:

$$q_e = K_F c_e^{1/n} \text{......................................} \quad (S6)$$

Where, K_F is the Freundlich constant; n is the exponent of adsorption strength, dimensionless.
